# Traditional practices and beliefs around placental disposal in Pakistan: Community perspectives and implications for environmentally safe and culturally sensitive practices

**DOI:** 10.12669/pjms.42.(ICON26).15691

**Published:** 2026-04

**Authors:** Mehreen Raza, Syeda Tabeena Ali, Sameera Ali Rizvi, Mah Talat

**Affiliations:** 1 Mehreen Raza, BDS, MBA (Healthcare Management), CHQP, Community Health Directorate, Indus Hospital and Health Network, Karachi - Pakistan; 2 Syeda Tabeena Ali, BSMT, MSPH, Community Health Directorate, Indus Hospital and Health Network, Karachi - Pakistan; 3 Sameera Ali Rizvi, MBBS, MSc Epidemiology and Biostatistics, Community Health Directorate, Indus Hospital and Health Network, Karachi - Pakistan; 4 Mah Talat, MBBS, MPH, Community Health Directorate, Indus Hospital and Health Network, Karachi - Pakistan

**Keywords:** Climate resilience, Cultural practices, Placenta disposal, Public health, Waste management

## Abstract

**Objective:**

This study explored the traditional beliefs, practices of the community and health system in relation to safe placental disposal in Pakistan, focusing on implications for environmental health and climate change.

**Methodology:**

A qualitative exploratory study was conducted from June 2025 to September 2025 at Primary Healthcare (PHC) centers of Indus Hospital and Health Network located in Sindh, Punjab, Baluchistan, and Kashmir. Data were collected through 14 in-depth interviews with women of reproductive age and three focus group discussions with midwives and the community health workers CHWs. All in-depth interviews (IDIs) and focus group discussions (FGDs) transcripts were created from verbatim data and then coded, triangulated with field notes, and thematically coded by following Braun and Clarke’s six-step framework for thematic analysis.

**Results:**

The five themes that emerged were knowledge and understanding of the placenta, cultural and symbolic beliefs, disposal practices, intergenerational roles, and cultural logic beyond hygiene. IDIs yield personal overtones such as the ideas of impurity, beliefs related to fertility, rituals and distrust of hospitals. FGDs highlighted shared consensus, where burial was a practice of honor and security and women were shown to be deprived of agency in hospitals.

**Conclusion:**

In Pakistan, placenta disposal is a culturally symbolic practice that has not been adequately incorporated into safe clinical protocols, which leaves gaps in environmental safety. The current challenges need culturally sensitive, environmentally sustainable and climate resilient approaches in healthcare systems.

## INTRODUCTION

Placenta, an essential organ in the formation of fetus, is not just a biological organ, but holds cultural, spiritual, and symbolic significance in most societies globally. The handling and disposal of placenta is a common practice in childbirth rituals in several low-and middle-income countries (LMICs). Culturally, these traditions differ in regions, religions and ethnic groups and reflect the perceived role of placenta in the health of mother and newborn; specifically in promoting fertility and avoiding spiritual damage.^1^,^2^ Most societies hold that placenta is the carrier of some portion of the child’s soul and poor disposal can lead to disease or bad luck.^3^ Burial with prayers/ religious recitation is the most popular practice in Muslim societies.^4^

Placenta disposal guidelines state that it must be kept cool and in a container with a seal, and disposed of within 48-72 hours. Placenta should be burned at high temperatures or buried at adequate depths, as opposed to being discarded as garbage. This may be subject to local laws and safe handling is crucial to avoid environmental contamination.^5^

Small facility-based births in LMICs begin to perceive placenta as clinical waste, which becomes at odds with the social norms among communities. This led to distrust of hospitals, with some families not willing to give birth in a hospital that does not allow placenta retrieval for traditional disposal.^5^,^6^ Poor handling of placental wastes is an environmental challenge to health system resilience in Pakistan. Waste management standards set by the national and provincial authorities demand a regulated disposal,^6^ but enforcement, poor segregation, and practice of relying on informal methods have created gaps and heightened the risk of soil and water contamination, particularly in flood-prone areas that exhaust sanitation infrastructure.^7^

Worldwide, the World Health Organization (WHO) determines healthcare waste management to be central to climate-resilient health facilities.^8^ Poor disposal of anatomical waste has been associated with environmental and health hazards.^8^,^9^ In Pakistan, traditional practices contribute to soil and water contamination, attraction of animals, and potential spread of infections, reducing the climate resilience of local communities.^10^

The study aimed to discuss the cultural, symbolic and social significance of the placenta and how the traditional disposal practices intersect with environmental and climate-related issues.

## METHODOLOGY

A qualitative exploratory study was conducted using IDIs and FGDs. Women of reproductive age between 18 and 49 years, coming to the selected PHC center and midwives and CHWs who were working at the same centers. Verbal and written informed consent were obtained for IDI and FGDs, respectively. All information was kept confidential by assigning the unique participant codes and recording the information in password-secured files, which were only accessed by the research team.

### Ethical approval:

It was obtained from the IHHN Institutional Review Board, bearing number IHHN_IRB_2025_07_011; dated August 15, 2025.

### Inclusion Criteria:

- Women of reproductive age between 18 and 49 years, with at least one childbirth, coming to the selected PHC center were included in the IDIs.
- Midwives and CHWs who were working at the same centers for at least one year were included in the group discussions.

### Exclusion Criteria:

- Women who were unwilling to participate were excluded.

### Data Collection:

The study was conducted from June to September 2025 at the PHC centers of IHHN located in Sindh, Punjab, Baluchistan, and Kashmir. The fourteen one-on-one IDIs and three FGDs were conducted using a purposive sampling approach to select participants according to eligibility criteria. A total of 41 participants were selected. IDIs were carried out with women from the community who while FGDs were conducted with midwives. Data was collected using an open-ended pretested interview guide to ensure the validity of the data collection tool. After taking participants’ consent, interviews were conducted in Urdu or the local language by research team. Detailed field notes were taken, and all interviews were audio-recorded. Later, all the verbatim data through audio recordings were transcribed, data was coded, and transcriptions were entered into Excel for data organization and coding.

Transcriptions were used to identify codes, sub-codes, and themes, which were converted into categories triangulated with field notes and thematically coded. Data was analyzed manually using Braun and Clarke’s six-step framework for thematic analysis.

## RESULTS

Thematic analysis of 14 IDIs and three FGDs showed that the practice of placental disposal is deeply rooted in cultural practices and symbolic beliefs across different provinces (Table-I). The IDIs gave individual-level narratives which emphasized personal experiences, uncertainties and symbolic differences; the FGDs presented collective present-day practices concerning cultural beliefs and healthcare practices. Both approaches combined emphasize five general themes. The themes and subthemes identified from IDIs and FGDs are presented in Table-II.

**Table-I:** Regional Profile of Study Participants.

Method	Region	n	Participants
FGDs	Sindh	8	Midwives & CHWs
	Baluchistan	7	
	Punjab & Kashmir	12	
IDIs	Sindh	4	Women of reproductive age 18–49 years
	Baluchistan	10	

***Note:*** IDIs were not conducted in Punjab & Kashmir due to weather constraints during the monsoon season, which limited field access and participant availability.

**Table-II:** Thematic Analysis of Placenta Disposal Practices (IDIs & FGDs).

Theme	Sub-themes	Codes
Knowledge & Understanding	Awareness and knowledge gaps	Impure yet life-giving Baby’s covering/sac Elders give meaning No hospital information Unaware of significance
Cultural & Symbolic Beliefs	Burial as symbol of respect Fertility and rituals Harm avoidance	Maternal dignity Burial linked with honour Believed to affect fertility Ritual markers of life Harm or bad luck if exposed
Disposal Practices	Home delivery Healthcare practices Mistrust in hospitals	Cloth wrapping Burial at home Placenta as waste Hospital mistrust Fear of misuse
Intergenerational Roles	Role of elders Continuity and lack of agency	Elders/dais responsible Maternal weakness Tradition continued Women lack agency
Beyond Hygiene	Tradition over Hygiene	Cultural logic Respect over hygiene Tradition over environment

### Theme-1: Knowledge and Understanding of the Placenta:

Both IDIs and FGDs showed limited knowledge about the placenta.

*“They say it is impure, but it was something that kept the baby alive inside me.”* (FGD - Baluchistan) *“After my delivery in the hospital, I don’t know what happened to it. Nobody explained, and I didn’t ask.”* (IDI - Sindh) *“I knew it only as something to be thrown away, but elders told us it holds meaning.”* (IDI - Punjab)

### Theme-2: Cultural and Symbolic Beliefs:

Disposal practices were based largely on cultural beliefs. such as the direction of the burial, facing downward or upward, have an impact on fertility. Proper coverage of the burial site with wood indicated a secure burial. FGDs emphasized burial as an aspect of dignity and security, whereby poor burial was linked to misfortune.

*“Our dai tells us: if you bury it facing up, more children will be born soon; if you bury it facing down, the next birth will be delayed.”.”* (IDI - Baluchistan) *“The norm of the community is that if the placenta is left exposed, it can bring bad luck to the family so it must be buried immediately.”* (FGD - Punjab) *“It is understood by women that placenta is part of the mother’s body - her honor. Throwing it away is like throwing away her dignity.”* (FGD - Sindh)

### Theme-3: Placenta Disposal Practices:

In both IDIs and FGDs showed mistrust of hospital practices where placentas were treated as waste.

*“At home, we wrap it with cloth and bury it because that is how life is respected.”* (IDI - Baluchistan) *“In the hospital, they toss it aside like rubbish, but for us it carries meaning that cannot be discarded.”* (IDI - Punjab) *“Some families insist on taking it home; they fear misuse, like feeding it to animals.”* (FGD - Sindh)

### Theme-4: Intergenerational Roles:

Interviews showed that elders, especially dais and grandmothers, were considered as being responsible for managing the placenta

“*The mother cannot touch it; she is weak. The elders, the mother-in-law or the dai, do what must be done*.” (IDI – Sindh) “*This is how our grandmothers did it; we continue without question*.” (IDI – Punjab) “*At the center, women were not bothered about the fate of the placenta and they never asked for it.”* (FGD – Kashmir)

### Theme-5: Cultural Logic Beyond Hygiene:

Placental disposal was a cultural practice and the participants seldom associated disposal with hygiene, sanitation or environmental considerations.

*“We never thought of health or environment; we only thought of following the way our elders taught us.”* (IDI - Baluchistan) *“This is not about hygiene or environmental contamination. It is about respect and continuing what has always been done.”* (FGD - Sindh) *“People here don’t consider sanitation; they speak of tradition.”* (FGD - Punjab).

## DISCUSSION

This qualitative research investigated traditional practices, symbolism and community perception in relation to placenta disposal in Pakistan. Findings indicate that placental disposal is heavily connected to ideas of dignity, maternal honor and protection, although placenta is perceived both as essential and impure. Burial was the socially accepted practice and intergenerational continuity occurred.

Building upon these findings, similar beliefs have been reported in other LMICs where the placenta carried deep spiritual and symbolic significance. Communities in Ethiopia explained the practice of placenta disposal associated with child protection and avoidance of spiritual evil and burial was the most respectful process^11^. Globally, it has been found that the disposal ritual among South Asian communities is mostly linked to fertility, identity and symbolic continuity^12^. Within the framework of Indigenous Australian people, females focused on placenta as cultural heritage and land membership and placenta gardens became symbolic practices of continuity^3^,^13^. These global similarities highlight the significance of placenta as a biological waste and represent cultural identity and maternal respect in every setting.

In Pakistan, such beliefs continue to shape local practices. Improper disposal by the community was also identified; participants often feared that it might *bring bad luck* in case if the placenta is not buried. As one participant shared,

*“The norm of the community is that if the placenta is left exposed, it can bring harm or bad luck to the family, which is why it must be buried immediately.”* (FGD - Punjab)

These perceptions reflect findings from other South Asian and African contexts. Studies from Somalia and Ethiopia indicated that exposing placenta to air was linked to sickness or magical harm.^11^,^14^ Likewise, in India, the placenta is viewed as ritually polluted, and specific disposal rituals are practiced to restore purity and ensure spiritual protection.^15^

Placenta is considered as clinical waste in healthcare institutions. Similarly, in Nigeria placenta is disposed using the hospital’s waste system, a practice that in many instances is in direct opposition to the family’s desires to recover the placenta to carry out their rites.^16^ In health facilities of Pakistan, UNICEF WASH assessment identified variation in placental waste handling and highlighted gaps in safe disposal practices. Similar to this study, the disjuncture caused mistrust and some of the families did not want institutional deliveries as it didn’t safeguard their tradition.^17^,^18^ Besides cultural and institutional implications, environmental and climate-resilient dimensions are also to be considered. Improper disposal practices, such as discarding in open space and or near a water source, can lead to contamination and attract animals, therefore increasing environmental and health hazards.^19^

Sustainable and climate-resilient health systems recommended by the World Health Organization should include the safe handling of anatomical waste, such as placentas, to significantly reduce environmental risks. New climate-intelligent technologies like biodigesters and non-burning technologies are suggested as viable alternatives.^20^,^21^

Hospital placenta disposal policies should respect cultural beliefs, build trust through staff training to promote safe, climate-resilient practices.

### Limitations and Strengths:

Participants were included from diverse regions of Pakistan, Sindh, Baluchistan, Punjab and Kashmir.

This study may have information bias due to facility-based sampling and language barriers. Moreover, there is limited research from Pakistan on placenta disposal practices which restricts local comparison.

## CONCLUSION

Placental disposal in Pakistan is well embedded in cultural practices of honor and protection which may contradict hospital practices that identify it as biomedical waste. This leads to mistrust health facilities and neglects environmental threats as well as the health protection of the citizens. Culturally sensitive strategies aligning with community norms need to be devised to strengthen clinical waste disposal practices and develop climate-resilient systems. This will earn the confidence of communities in the healthcare systems and safeguard environmental health.

### Authors Contribution:

**MR** Conceptualization, data collection

**SAR** Study methodology, Result interpretation and responsible for the integrity of the work.

**STA** Data analysis, critical review.

**MT** Critical review of manuscript, literature search

All authors have approved the final version of the manuscript.

## References

[1] Aynalem BY, Melesse MF, Bitewa YB (2023). Cultural beliefs and traditional practices during pregnancy, childbirth, and the postpartum period in East Gojjam Zone, Northwest Ethiopia:a qualitative study. Womens Health Rep (New Rochelle).

[2] Moeti C, Mulaudzi FM, Rasweswe MM (2023). The disposal of placenta among indigenous groups globally:an integrative literature review. Int J Reprod Med. 2023.

[3] McMullen N, Fiolet R, Redley B, Hutchinson AM (2024). A beautiful bush space on Country:Indigenous women's perspectives on the cultural significance of a placenta garden. Women Birth.

[4] Hassan SM (2022). Religious practices of Muslim women in the UK during maternity:evidence-based professional practice recommendations. BMC Pregnancy Childbirth.

[5] Heller DS, Faye-Petersen O, Baergen RN, Kaplan C (2009). Handling of perinatal specimens:a Society for Pediatric Pathology practice committee survey. Pediatr Dev Pathol.

[6] Carreon CK, Ravishankar S, Parast MM, Castro EC, Baergen RN, Bonasoni MP (2023). Releasing placentas to families:a unified recommendation from the Perinatal Committee of the Society for Pediatric Pathology. Arch Pathol Lab Med.

[7] Hussainzad EA, Gou Z (2024). Climate risk and vulnerability assessment in informal settlements of the Global South:a critical review. Land.

[8] World Health Organization (2020). Climate-resilient and environmentally sustainable health care facilities. https://www.who.int/teams/environment-climate-change-and-health.

[9] Janik-Karpinska E, Brancaleoni R, Niemcewicz M, Wojtas W, Foco M, Podogrocki M (2023). Healthcare waste—a serious problem for global health. Healthcare (Basel).

[10] UNICEF Pakistan (2021). Scoping study to establish a baseline for reporting to SDGs for WASH in health care facilities in Pakistan. https://www.unicef.org/pakistan/media/5226.

[11] Ahmed M, Demissie M, Worku A, Abrha A, Berhane Y (2019). Socio-cultural factors favoring home delivery in Afar pastoral community, northeast Ethiopia:a qualitative study. Reprod Health.

[12] Felisian S, Mushy SE, Tarimo EA, Kibusi SM (2023). Sociocultural practices and beliefs during pregnancy, childbirth, and postpartum among indigenous pastoralist women of reproductive age in Manyara, Tanzania:a descriptive qualitative study. BMC Womens Health.

[13] Thackrah RD, Wood J, Thompson SC (2020). Cultural respect in midwifery service provision for Aboriginal women:longitudinal follow-up reveals the enduring legacy of targeted program initiatives. Int J Equity Health.

[14] NHS Wales (2025). Placenta examination, histological investigation and disposal of placenta following birth guideline. https://wisdom.nhs.wales/health-board-guidelines/.

[15] Wells YO, Dietsch E (2014). Childbearing traditions of Indian women at home and abroad:an integrative literature review. Women Birth.

[16] Nnagbo JE, Ugwu GO, Eze MI, Agu PU, Nnagbo CL, Nkwo PO (2022). Placenta disposal practices among doctors and nurses in obstetric units of secondary and tertiary health facilities in Enugu State, Nigeria. Niger J Med.

[17] Tegegne KT, Wudu TK, Abdisa BG, Tegegne ET, Tessema MK (2023). Institutional delivery knowledge, attitude, and practice among mothers of childbearing age with one or more children, Ethiopia. J Prev Med Hyg.

[18] Tarik YD, Nigussie AA, Balcha WF, Getu AA (2022). Factors associated with institutional delivery among mothers who gave birth within 1 year prior to the study at Gilgelbelles town, Northwest Ethiopia:a mixed-methods study. BMJ Open.

[19] Daka E (2023). Adopting clean technologies to climate change adaptation strategies in Africa:a systematic literature review. Environ Manage.

[20] Mosadeghrad AM, Isfahani P, Eslambolchi L, Zahmatkesh M, Afshari M (2023). Strategies to strengthen a climate-resilient health system:a scoping review. Glob Health.

[21] Fatimi AS, Mahmud O (2022). The costs of protecting health in the face of climate change –feasibility lies in proactivity. J Pak Med Assoc.

